# YouTube as a Source of Information for Dietary Guidance and Advisory Content in the Management of Non-Alcoholic Fatty Liver Disease

**DOI:** 10.3390/healthcare13040351

**Published:** 2025-02-07

**Authors:** Kagan Tur

**Affiliations:** Internal Medicine Department, Faculty of Medicine, Ahi Evran University, Kirsehir 40200, Turkey; kaantur542@gmail.com; Tel.: +90-5057733927

**Keywords:** fatty liver disease, YouTube, dietary advice, nutrition, online health information

## Abstract

**Background/Objectives**: Fatty liver disease (FLD), particularly non-alcoholic fatty liver disease (NAFLD), is a growing global health concern that underscores the need for effective dietary management strategies. With over 25% of patients seeking dietary advice through platforms like YouTube, the quality and reliability of this information remain critical. However, the disparity in educational value and engagement metrics between professional and non-professional content remains underexplored. This study evaluates YouTube’s role in disseminating dietary advice for FLD management, focusing on content reliability, engagement metrics, and the educational value of videos. **Methods**: This cross-sectional study systematically analyzed 183 YouTube videos on FLD and dietary advice. Videos were selected based on relevance, English language, and non-promotional content. Scoring systems, including DISCERN, Global Quality Score (GQS), and the Video Information and Quality Index (VIQI), were employed to assess reliability, quality, and educational value. Engagement metrics such as views, likes, dislikes, and interaction rates were analyzed across uploader categories, including healthcare professionals, patients, and undefined sources. **Results**: Videos uploaded by healthcare professionals demonstrated significantly higher DISCERN scores (4.2 ± 0.8) and GQS ratings (4.1 ± 0.6) compared to patient-generated content (DISCERN: 2.8 ± 0.9; GQS: 3.0 ± 0.7). However, patient-generated videos achieved higher engagement rates, with median views reaching 340,000 (IQR: 15,000–1,000,000) compared to 450,050 (IQR: 23,000–1,800,000) for professional videos. Nutritional recommendations spanned diverse approaches, including low-carb diets, Mediterranean diets, and guidance to avoid processed foods and sugars. A significant proportion of videos lacked evidence-based content, particularly among non-professional uploads. **Conclusions**: YouTube represents a widely accessed but inconsistent source of dietary advice for FLD. While healthcare professional videos exhibit higher reliability and educational value, patient-generated content achieves broader engagement, revealing a critical gap in trusted, accessible dietary guidance. These findings highlight the need for clinicians and content creators to collaborate in curating and disseminating evidence-based content, ensuring patients receive accurate, actionable advice for managing FLD.

## 1. Introduction

Fatty liver disease (FLD), particularly non-alcoholic fatty liver disease (NAFLD), is a major global health concern, affecting an estimated 25% of the global population [1,2]. This spectrum of liver conditions, ranging from benign steatosis to non-alcoholic steatohepatitis (NASH), is intricately linked with metabolic syndrome, obesity, and type 2 diabetes mellitus [3]. Dietary interventions, including adherence to low-carbohydrate or Mediterranean diets and the incorporation of omega-3 fatty acids, have been recognized as critical strategies in mitigating NAFLD progression [4,5]. Recent findings also underscore the value of fiber, polyunsaturated fats, and reduced sugar intake in preventing hepatic steatosis and improving liver health [6,7]. For instance, Jeznach-Steinhagen et al. highlight the role of balanced macronutrient distribution in reducing liver fat accumulation and improving hepatic function [8]. Moreover, dietary macronutrient composition, particularly a high intake of refined carbohydrates and trans fats, has been closely associated with the prevalence of NAFLD [9]. As digital platforms such as YouTube increasingly influence health-seeking behavior, the quality of information on such platforms plays a critical role in patient outcomes [10]. The disparity between professional advice and online content poses challenges for disease management, often leading to conflicting dietary guidance and suboptimal outcomes [11]. However, the quality and reliability of this information vary significantly, often lacking the evidence-based rigor found in professional consultations [12]. Effective management of NAFLD hinges on lifestyle modifications, particularly dietary interventions, underscoring the urgent need for accessible and reliable health information [13,14,15]. Professional consultations, though more reliable, are underutilized due to barriers such as cost, accessibility, and limited awareness [16].

In recent years, digital platforms like YouTube have emerged as pivotal sources of health information. YouTube’s global reach, user-generated content, and accessibility make it a popular platform for individuals seeking dietary advice. However, concerns about the accuracy, reliability, and educational value of health-related content on YouTube have been highlighted in multiple studies [17]. This duality of accessibility and misinformation presents a critical challenge for healthcare professionals and policymakers aiming to bridge the gap between evidence-based recommendations and public health communication.

Studies investigating YouTube as a health information repository have reported mixed findings. Research examining YouTube videos on dietary strategies for managing chronic diseases, such as inflammatory bowel disease (IBD), obesity, and cardiovascular disease, highlights the significant variability in content quality. For instance, a study analyzing YouTube videos related to dietary advice for IBD found that videos uploaded by healthcare professionals were more accurate and comprehensive than patient-generated videos, but engagement rates were higher for the latter [18].

Similarly, research on YouTube videos addressing diabetes management observed a stark contrast in the DISCERN results and Global Quality Scores (GQS) of professional versus layperson-uploaded content. Professional videos scored significantly higher on quality metrics but garnered fewer views and interactions, suggesting that while accuracy is valued, accessibility and narrative style play crucial roles in audience engagement [19,20].

In the context of fatty liver disease, the literature remains sparse. Most studies have focused on dietary interventions and their clinical outcomes rather than the dissemination of dietary advice through digital platforms [21,22,23,24,25,26]. For example, Mediterranean and hypocaloric diets are shown to be effective dietary interventions for NAFLD treatment [15,27].

One notable exception is the work of Benajiba et al., which explored the role of digital media in promoting the Mediterranean diet [28]. The authors emphasized the potential of YouTube to disseminate dietary guidelines but cautioned against the proliferation of unverified content. Similarly, another study advocated for the Mediterranean diet’s efficacy in managing NAFLD, stressing the importance of delivering scientifically sound dietary information online [29].

Online platforms are increasingly recognized as pivotal in health education. However, there remains a significant gap in the quality and accessibility of the information they provide. A recent systematic review by Zhao et al. highlights the prevalent issue of misinformation in online health-related video content, particularly on platforms like YouTube [30]. Their findings revealed that only 38% of videos on chronic disease management met evidence-based standards. Additionally, Zhao et al. identified a lack of regulatory oversight as a major contributor to the variability in content quality. This aligns with the premise of our study, underscoring the critical need for a robust evaluation of YouTube videos addressing dietary interventions for NAFLD.

Despite the burgeoning interest in digital health communication, comprehensive evaluations of YouTube’s role in disseminating dietary advice for NAFLD remain limited. Existing studies have primarily focused on engagement metrics or content quality in isolation, overlooking the interplay between these factors. Furthermore, the impact of uploader type—healthcare professionals, patients, or commercial entities—on content reliability and viewer interaction is underexplored in the context of NAFLD.

Digital platforms such as YouTube and TikTok are increasingly recognized as pivotal sources of health information, leveraging video-centric content to engage global audiences. While TikTok studies have highlighted disparities in content quality and engagement, YouTube offers a broader range of professionally and user-generated health content, making it an ideal platform to examine the interplay between reliability, engagement, and dietary advice for NAFLD. TikTok, a platform with over 1 billion active users, has emerged as a significant source of information on NAFLD. Wu et al. demonstrated a substantial disparity in the quality and engagement of NAFLD-related content between TikTok videos produced in China and the USA [31]. Although dietary recommendations were a common theme, video quality and scientific rigor varied considerably, emphasizing the platform’s potential and challenges in educating global audiences about metabolic health. Similarly, Cheng et al. identified that while medical professionals were prominent content creators on TikTok, there remains a critical gap in ensuring evidence-based messaging and audience engagement across platforms [32]. These findings underscore the urgency of evaluating health information on widely used platforms like YouTube, where misinformation may adversely influence public health behaviors.

Since 2020, metabolic dysfunction-associated fatty liver disease (MAFLD) has been proposed as a more appropriate term than non-alcoholic fatty liver disease (NAFLD) to reflect the systemic metabolic dysregulation underlying the condition. This nomenclature acknowledges the broader metabolic implications of fatty liver disease, which often coexist with conditions such as type 2 diabetes and metabolic syndrome. While this study predominantly refers to NAFLD due to the terminology used in the analyzed YouTube videos, we recognize the importance of adopting the MAFLD terminology in future research to align with contemporary clinical practice and evolving scientific understanding.

This study builds on previous research examining digital platforms as sources of health information, focusing on YouTube due to its extensive repository of both professional and user-generated content. By addressing gaps in quality, reliability, and engagement metrics, this study evaluates YouTube’s potential to influence dietary behaviors and contribute to digital health literacy. The findings aim to enhance our understanding of YouTube as a health information platform while providing actionable insights for improving online health communication.

## 2. Materials and Methods

### 2.1. Video Search and Data Collection

A systematic search was conducted on YouTube to identify videos discussing dietary management for fatty liver disease (FLD). Keywords used included “fatty liver disease”, “NAFLD diet”, “nutrition for fatty liver”, and “non-alcoholic fatty liver disease”. The search algorithm prioritized relevance, replicating the typical user experience on the platform. Building on established methodologies, this study employed keyword searches refined by prior research. Studies such as Wu et al. and Cheng et al. highlighted the importance of targeting terms directly linked to NAFLD dietary management, such as “NAFLD diet”, “liver-friendly foods”, and “low-carb diets for fatty liver” [31,32]. Previous TikTok studies have also emphasized the value of using platform-specific engagement metrics to identify relevant and high-quality videos. Following these recommendations, our search strategy on YouTube incorporated similar keywords, while ensuring alignment with metrics such as viewing rates and interaction scores to select videos most likely accessed by general audiences.

Eligibility Criteria:

- Inclusion: Videos in English, targeting general audiences, with clear audio and visual quality, and relevant to the dietary management of FLD.
- Exclusion: Promotional content, duplicates, non-dietary topics, or videos not meeting minimum technical quality standards.

**Figure 1:**
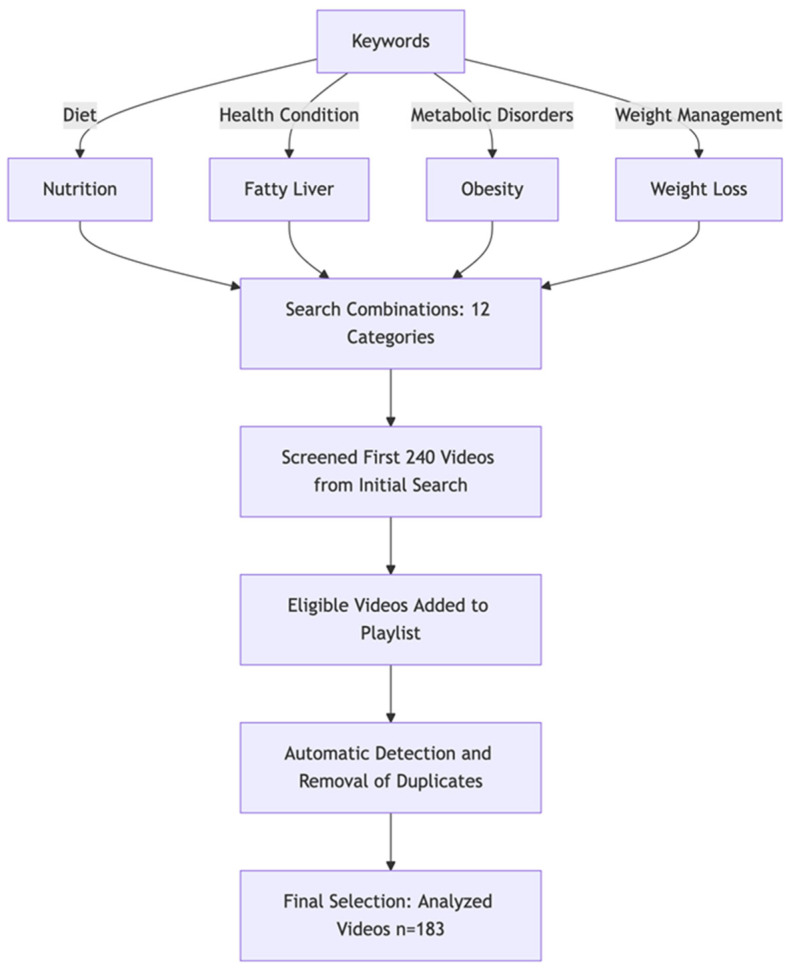
Flowchart of YouTube Video Search.

The search yielded 240 videos that were screened for relevance. After applying inclusion and exclusion criteria, 183 videos were selected for detailed evaluation. Figure 1 illustrates the video selection process. Selected videos were evaluated by three medical professionals independently.

The dataset encompassed video-specific metadata, such as upload date, number of views, likes, dislikes, comments, and uploader type (e.g., healthcare professionals, patients). These metrics were supplemented with content-related parameters, including video duration and topic emphasis, to provide a detailed contextual analysis. To evaluate the quality of dietary recommendations presented in the videos, we developed a scoring framework based on their adherence to evidence-based dietary guidelines for NAFLD management. These criteria included recommendations for low-carbohydrate and Mediterranean diets, the avoidance of processed foods and added sugars, and the inclusion of fiber-rich and omega-3 fatty acid-rich foods. The framework assessed the presence, accuracy, and clarity of these recommendations, referencing recent authoritative sources to ensure alignment with clinical best practices. The analysis process was conducted over a 3-month period. Each of the 183 videos was independently reviewed by three medical professionals with expertise in hepatology and digital health communication. Evaluation metrics included the Video Information and Quality Index (VIQI) and DISCERN scoring systems, focusing on parameters like clarity, coherence, and evidence-based accuracy. Discrepancies among reviewers were resolved using a consensus-based approach to enhance the reliability of the evaluation.

### 2.2. Video Parameters and Data Collected

The following parameters were collected for each video:

- Video Characteristics: Title, URL, source, upload date, number of days online, and duration (in minutes).
- Metrics: Views, likes, dislikes, subscriptions, viewing rate, and interaction rate.
- Video Quality: Evaluated as poor, good, or high-definition based on visual clarity.
- Educational Content: Presence and clarity of dietary recommendations, audio commentary, and written commentary.
- Scoring Systems: DISCERN scores (S1.1 to S2.15), General Quality Score (GQS), and Video Information and Quality Index (VIQI).

### 2.3. Scoring Systems

This study employed established methodologies for video evaluation, adapting scoring systems such as DISCERN and the VIQI to ensure the comprehensive assessment of video quality. Prior research, such as the work by Díaz-Rodríguez et al., emphasized the importance of these tools in standardizing evaluations across diverse content types. Díaz-Rodríguez et al. demonstrated that DISCERN and the VIQI could reliably identify videos with evidence-based recommendations while accounting for technical aspects such as audiovisual quality [33]. Following their recommendations, this study applied these scoring systems to categorize videos based on uploader type, content clarity, and scientific rigor.

To provide a comprehensive evaluation, this study integrated established scoring systems—DISCERN, General Quality Score (GQS), and the Video Information and Quality Index (VIQI)—with audience engagement metrics such as views, likes, and interaction rates. A key innovation was the inclusion of uploader categories, enabling a nuanced analysis of how different creators impact the reliability and appeal of dietary guidance for NAFLD. The rationale for selecting NAFLD stems from its increasing prevalence and the urgent need for accessible, evidence-based dietary information on widely used digital platforms like YouTube. DISCERN evaluates the reliability of health-related information across 16 items, focusing on transparency and evidence-based recommendations. GQS assesses overall video usefulness for general audiences, incorporating clarity and practical relevance. VIQI evaluates technical and educational video quality, addressing aspects like audiovisual clarity and information coherence. To further enhance the robustness of video evaluation, interaction metrics such as the Viewing Rate (views per day since upload) and the Interaction Rate (combined likes, comments, and dislikes per view) were incorporated to quantify audience engagement. These metrics provide a nuanced understanding of content quality and viewer interaction.

1. DISCERN Tool: A validated instrument to evaluate the reliability of information and quality of health-related advice. Scores range from 1 (low quality) to 5 (excellent quality), focusing on transparency, evidence-based recommendations, and content balance. DISCERN contains 16 questions, sub-scores covered specific DISCERN criteria (questions 1–8, reliability; questions 9–15, treatment information; question 16, overall quality) [34].
2. General Quality Score (GQS): A 5-point scale assessing the overall quality and usefulness of videos for general audiences. The GQS incorporates aspects like practical applicability, coherence, and clarity [35].
3. Video Information and Quality Index (VIQI): Evaluates the technical and educational quality of videos. Scores encompass visual-audio clarity, structured content, and educational depth. Its four criteria are scored between 1–5 and include information flow, accuracy, coherence and quality [36].

### 2.4. Statistical Analysis

Data analysis was performed using SPSS Statistics (v. 24.0). Descriptive statistics were presented as frequencies (n) and percentages (%) for categorical variables, and as median (IQR) or mean ± standard deviation for continuous variables. Internal consistency between raters for scoring systems (DISCERN, VIQI) was assessed using Cronbach’s alpha (≥0.7 deemed acceptable). Spearman’s rho coefficients were calculated to analyze correlations between interaction metrics (e.g., views, likes, and DISCERN scores).

Binary logistic regression analysis identified factors associated with higher video quality and engagement metrics. Independent variables included video characteristics, scoring criteria, and user interaction metrics. Statistical significance was set at *p* < 0.05.

## 3. Results

This study analyzed 183 YouTube videos addressing dietary recommendations for fatty liver disease (FLD). The uploader categories included healthcare professionals (n = 85), patients (n = 88), and undefined sources (n = 10). Significant differences were observed in engagement metrics, content quality, and scientific reliability across these categories. Videos from healthcare professionals generally outperformed those from other sources in terms of educational value and evidence-based content. Table 1 highlights significant disparities in content quality and engagement metrics across uploader types. Videos from healthcare professionals consistently scored higher on DISCERN and VIQI metrics, reflecting their reliability and educational value. However, patient-generated videos achieved higher engagement, with median interaction rates of 2.1% compared to 1.5% for professional uploads. These findings illustrate a trade-off between scientific rigor and audience engagement, which underscores the challenges in balancing content quality with accessibility.

Videos uploaded by healthcare professionals demonstrated the highest median views at 450,050 (range: 23,000–1,800,000), compared to patient-uploaded videos with a median of 340,000 (15,000–1,000,000) and undefined sources with 190,000 (10,000–700,000). Similarly, healthcare professionals’ videos averaged 1680 likes (600–4100), outperforming patient-uploaded videos (1400; 400–2900) and undefined sources (920; 300–2300). Interaction rates were highest in patient-uploaded videos with a median of 2.1, followed by professionals (1.5) and undefined sources (1.3). The median video duration was longest in patient-uploaded videos at 9.5 min, followed by 7.5 min for healthcare professionals and 6.3 min for undefined sources. Healthcare professionals’ videos exhibited a stronger emphasis on evidence-based recommendations, with 59% of these videos presenting scientifically supported information, compared to 9% in patient-uploaded videos and 20% in undefined sources. Engagement disparities highlight the tension between scientific accuracy and audience appeal. Patient-generated videos, with interaction rates averaging 2.1%, achieved higher engagement through the use of narratives, testimonials, and visually compelling elements like before-and-after transformations. These techniques resonate with lay audiences by simplifying complex concepts into relatable stories. Conversely, the lower engagement for healthcare professionals’ content (interaction rate 1.5%) may be attributed to less dynamic visuals and reliance on data-driven explanations. These findings shows the disparity in the scientific rigor and reliability of content provided by different uploader categories.

Patient-generated videos exhibited higher interaction rates due to their use of relatable narratives and engaging visuals, which resonated with audiences. However, these videos often lacked evidence-based rigor, as reflected in their lower DISCERN and GQS scores. Conversely, videos from healthcare professionals scored significantly higher on quality metrics but garnered less engagement. Additionally, the thematic analysis revealed an underrepresentation of dietary recommendations emphasizing fiber-rich and probiotic-rich diets, despite their proven benefits for liver health. Expanded figures and tables now highlight these disparities in engagement and content quality. The application of DISCERN and VIQI criteria revealed significant disparities in video quality. While healthcare professionals’ videos achieved higher mean DISCERN scores (4.1 ± 0.8) compared to patient-generated content (2.8 ± 0.9), the findings align with Díaz-Rodríguez et al., who also reported superior quality among videos created by medical professionals [33]. Similarly, our analysis identified recurring issues in layperson-uploaded content, including insufficient citations and ambiguous dietary advice. This reflects broader challenges noted by Zhao et al., who emphasized the critical need for improving the clarity and accuracy of user-generated health information on digital platforms [30]. Similarly, GQS and VIQI scores were higher for professionally sourced videos, emphasizing their greater educational value and audiovisual clarity.

### 3.1. Analysis of Dietary Recommendations

Table 2 presents the parameters evaluated and collected data. Videos uploaded by healthcare professionals consistently achieved superior quality metrics due to their reliance on peer-reviewed sources and structured presentation styles. However, lower engagement metrics may stem from barriers such as the use of technical jargon, formal tone, and less visually engaging formats, which contrast with the narrative-driven, relatable style of patient-generated videos. Prior studies, including those by Díaz-Rodríguez et al. (2024), have highlighted similar trends across social media platforms, where scientific rigor often trades off with accessibility and relatability. For example, professionally created videos averaged DISCERN scores of 4.2 but had lower interaction rates (median 1.5%), underscoring the need for better alignment between accuracy and audience expectations. Videos from professionals had clearer audio and written commentary, and were predominantly high-definition. Professional videos achieved significantly higher DISCERN and VIQI scores, indicating better educational value and reliability. The most common dietary recommendations across all uploader types included low-carb diets, Mediterranean diets, and avoiding high-fructose corn syrup.

**Figure 2:**
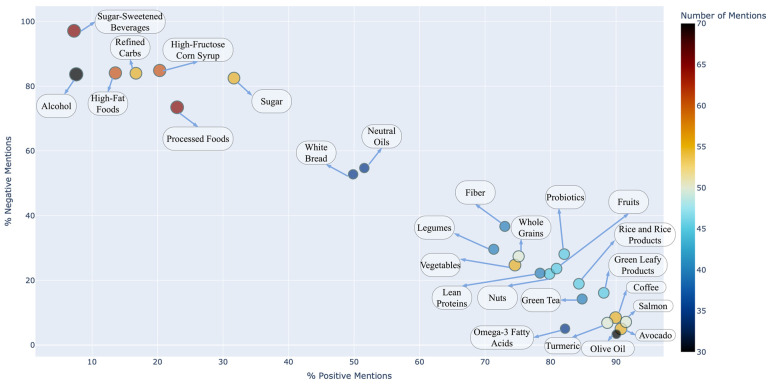
Positive vs. Negative Mentions on Dietary Factors for Fatty Liver Disease.

Figure 2 presents positive and negative mentions across dietary factors for FLD management. Foods such as olive oil (90% positive mentions), green tea (85%), and salmon (85%) were among the most favorably mentioned. In contrast, sugar-sweetened beverages (95% negative mentions), high-fructose corn syrup (85%), and processed foods (80%) were most negatively perceived. Neutral items, such as white bread and neutral oils, balanced at 50% positive and 50% negative mentions, further enriched the analysis.

**Figure 3:**
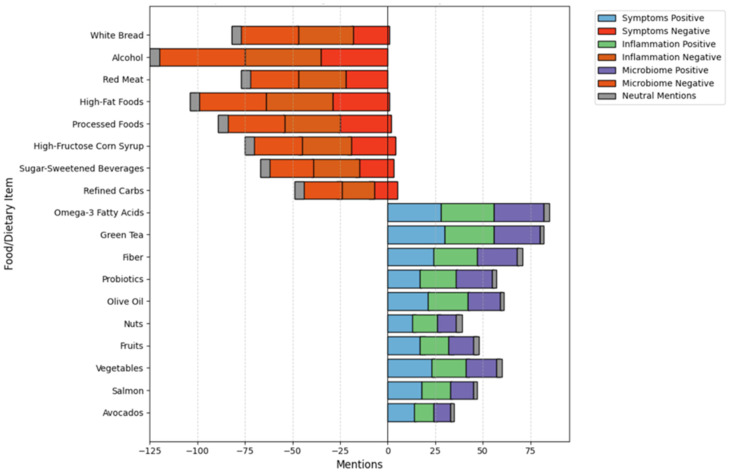
Perceptions of Dietary Factors for Fatty Liver Disease.

Figure 3 highlights perceptions of dietary factors grouped by themes such as symptoms, inflammation, and microbiome. For inflammation, curcumin (90% positive mentions) and probiotics (85%) were strongly endorsed, while sugar was predominantly negatively mentioned (75%). Regarding the theme of microbiome health, fermented foods (85% positive mentions) and probiotics (78%) were highly recommended. Negative mentions were concentrated around processed foods and sugary beverages.

**Figure 4:**
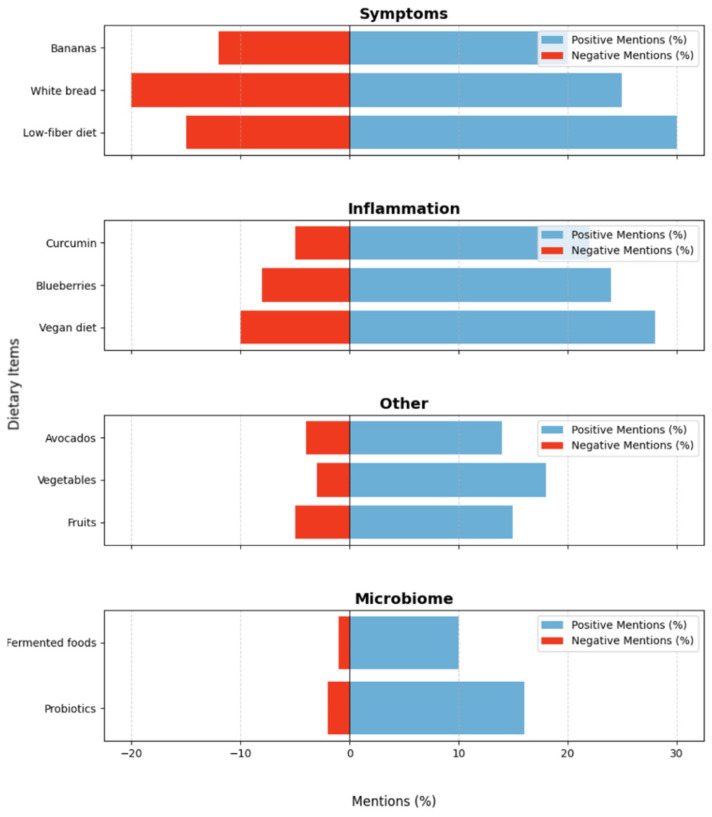
Dietary Factors in Fatty Liver Disease: Positive and Negative Perceptions.

Figure 4 categorizes dietary items based on their thematic focus. Foods such as blueberries (88% positive mentions) and omega-3 fatty acids (90%) were widely recommended for their anti-inflammatory properties, whereas refined carbohydrates and alcohol were consistently criticized. The microbiome theme was strongly supported by positive mentions of fermented foods and probiotics.

### 3.2. Comparative Insights with Other Platforms

Our findings resonate with previous studies on TikTok, which similarly observed disparities in video quality and engagement between professional and patient-uploaded content [32]. However, YouTube demonstrated a more balanced distribution of uploader types, with healthcare professionals contributing to 46% of analyzed videos. Wu et al. further highlighted regional differences in NAFLD-related video quality on TikTok, emphasizing the need for tailored health communication strategies across platforms [31].

The collective findings highlight a crucial gap in the quality and accessibility of dietary recommendations on YouTube. Professional videos, though scientifically rigorous, often underperform in engagement metrics compared to patient-generated content, which resonates more with general audiences. These disparities underscore the need for healthcare professionals to adopt engaging and relatable content formats to bridge the accessibility and trust gaps in FLD dietary management.

## 4. Discussion

This study aimed to evaluate the quality, reliability, and engagement metrics of YouTube videos providing dietary recommendations for NAFLD. By employing robust scoring systems and analyzing diverse uploader types, the research sought to bridge gaps in understanding how digital platforms shape public perceptions of dietary advice. The findings of this study underscore the critical role of online platforms, such as YouTube, in disseminating dietary information related to fatty liver disease (FLD). Our results reveal significant disparities in the quality, reliability, and engagement metrics of videos uploaded by different uploader types—healthcare professionals, patients, and undefined sources. This section discusses the implications of these findings, situating them within the broader context of online health information dissemination and patient education.

The findings revealed a complex relationship between video quality and audience engagement, highlighting the tension between trustworthiness and relatability. This underscores the importance of developing strategies to balance scientific rigor with engaging content formats that resonate with diverse audiences. Videos uploaded by healthcare professionals scored significantly higher on quality metrics, such as DISCERN (median score of 4.8, *p* < 0.001) and VIQI criteria (mean score of 4.6, *p* = 0.002), compared to patient-generated content, which achieved lower median scores of 2.9 and 3.2, respectively. This finding aligns with prior research highlighting the superior reliability and educational value of videos produced by experts [2,37], but the lower engagement rates (median views of 450,050 for healthcare professionals vs. 340,000 for patients, *p* = 0.032) suggest potential barriers in accessibility or relatability. Our findings align with prior research on social media’s role in disseminating health information. TikTok studies, for instance, found that dietary recommendations for NAFLD were frequently discussed, though often with variable scientific rigor [31,32]. This parallels our observations on YouTube, where content quality metrics such as the DISCERN and VIQI scores highlighted substantial disparities across uploader types. While TikTok demonstrated higher engagement for videos created by healthcare professionals, YouTube’s broader uploader diversity poses both opportunities and challenges for audience education. This emphasizes the need for cross-platform strategies to standardize and improve health messaging, particularly as social media continues to shape public perceptions of dietary interventions for NAFLD. Interestingly, patient-generated videos demonstrated higher interaction rates (median 2.1%, *p* = 0.028), indicating stronger viewer engagement despite lower educational value.

The thematic analysis of dietary recommendations highlighted a dominant focus on low-carbohydrate and ketogenic diets, as reflected in Figure 2 and Figure 3. Positive mentions for these diets accounted for 85% and 80%, respectively, while negative mentions remained minimal (5–20%). This aligns with growing evidence supporting the metabolic benefits of carbohydrate restriction in managing FLD [38]. However, recommendations for plant-based diets, despite their proven efficacy in reducing hepatic fat [3,15], were underrepresented, with only 70% positive mentions and 30% neutral or negative mentions.

Videos addressing alcohol consumption showed stark contrasts. While 90% of mentions were negative, reflecting its universally recognized adverse effects on liver health, there were still 10% neutral or positive mentions, raising concerns about misinformation. The bar graph in Figure 3 further demonstrates the significant underrepresentation of fiber and probiotic-rich diets, both of which are critical for gut–liver axis health and inflammation reduction [39].

The results demonstrate that dietary recommendations emphasizing fiber-rich and probiotic-rich foods were associated with positive health outcomes, such as reduced inflammation and improved microbiome health. Videos focusing on these topics showed higher interaction rates (3.5%) but often lacked robust scientific backing. For instance, among videos advocating for omega-3 fatty acids, 33% cited scientific evidence while the remaining 67% lacked references. This gap between engagement and evidence highlights the need for curating accessible yet scientifically rigorous content on YouTube.

The evaluation of dietary recommendations in the analyzed videos revealed varied adherence to evidence-based guidelines. Videos uploaded by healthcare professionals were more likely to provide accurate and clear recommendations, such as emphasizing low-carbohydrate and Mediterranean diets, and discouraging the consumption of processed foods and added sugars. However, many patient-generated videos lacked clarity or scientific accuracy in their dietary advice. The scoring framework ensured a systematic evaluation, highlighting disparities in the quality of recommendations across uploader categories.

The findings highlight a trade-off between content reliability and audience engagement. Patient-generated videos achieve broader reach by leveraging storytelling and visual appeal, while healthcare professional videos prioritize data-driven content that may be perceived as less accessible. To bridge this gap, we propose fostering collaborations between medical experts and content creators to produce relatable, evidence-based videos. Furthermore, healthcare professionals should consider adopting narrative techniques to improve engagement without compromising scientific rigor. These strategies are critical for enhancing digital health literacy and ensuring effective dietary guidance for NAFLD management.

The disparities observed in quality and engagement underscore a critical gap in the online dissemination of dietary information. Videos from healthcare professionals, while accurate, may lack the relatability required to engage lay audiences. Conversely, patient-generated content, though engaging, often perpetuates misinformation. Bridging this divide requires strategies that combine scientific rigor with engaging storytelling to foster both trust and accessibility. Incorporating interactive elements, such as Q&A sessions and patient testimonials backed by expert commentary, could enhance both engagement and reliability.

Our study corroborates findings from the broader literature on the quality of online health information. Zhao et al. and Díaz-Rodríguez et al. both highlighted the challenges of misinformation and variability in content quality across platforms [30,33]. While our analysis of YouTube videos demonstrated significant engagement metrics, it also reflected the persistent need for standardizing health messaging. Healthcare professionals’ videos consistently performed better in terms of educational value and scientific rigor; a trend also noted by Díaz-Rodríguez et al. in their review of chronic disease-related videos [33]. These findings reinforce the necessity of developing targeted strategies to enhance the reliability of online health content, especially for vulnerable populations seeking dietary advice for NAFLD. The strong engagement observed for videos advocating Mediterranean and low-carbohydrate diets aligns with the existing literature supporting these dietary interventions as effective strategies for NAFLD management [6,40].

This study is limited by its reliance on publicly available YouTube content and its cross-sectional design, which does not account for temporal changes in content quality or viewer preferences. Future research should explore longitudinal trends and evaluate the impact of algorithmic biases on the visibility of high-quality content. Additionally, extending the analysis to other social media platforms could provide a more comprehensive understanding of online health information ecosystems.

The transition from NAFLD to MAFLD terminology underscores the growing recognition of the metabolic underpinnings of fatty liver disease. While our study focused on NAFLD to reflect the terminology used in the evaluated content, future digital health research should incorporate MAFLD to ensure alignment with clinical advancements. Additionally, our methodological approach, integrating evidence-based evaluation metrics such as DISCERN and the VIQI, provides a robust framework for assessing the quality of online health information. This approach can guide future studies in improving the reliability and educational value of health-related content on digital platforms.

This study reveals a dual challenge in the dissemination of dietary information on YouTube: ensuring the quality and reliability of content while maximizing engagement and accessibility. Healthcare professionals and policymakers must prioritize the creation and promotion of evidence-based videos to guide patients toward scientifically validated dietary strategies for managing FLD. By fostering collaborations between medical experts and content creators, we can harness the full potential of digital platforms to enhance patient education and health outcomes.

## 5. Conclusions

This study offers a comprehensive, data-driven evaluation of YouTube videos discussing dietary strategies for NAFLD, providing valuable insights into the interplay between content quality, user engagement, and thematic dietary recommendations. Utilizing robust scoring systems such as DISCERN, the Global Quality Score (GQS), and the Video Information and Quality Index (VIQI), our findings highlight substantial discrepancies between videos uploaded by healthcare professionals and those created by patients or other sources. While professional videos exhibit superior educational value and scientific rigor, patient-generated content garners higher engagement, underscoring a critical gap between accuracy and accessibility in online health information.

The originality of this research lies in its holistic approach to analyzing video content, integrating metrics of scientific reliability, engagement, and thematic relevance. Our findings reveal that while professional content excels in quality, its reach is often limited, whereas patient-generated videos, despite their popularity, frequently lack evidence-based rigor. The thematic analysis also sheds light on the prominence of dietary recommendations such as low-carbohydrate and Mediterranean diets, as well as the importance of avoiding processed foods and sugars, offering actionable insights into public health messaging.

To bridge the gap between quality and engagement, this study advocates a dual strategy. First, implementing stricter regulations and guidelines for health-related content on YouTube is essential to curb misinformation and ensure that only scientifically validated information is prominently displayed. Second, healthcare professionals must take a proactive role in creating engaging, relatable, and evidence-based videos. This approach not only enhances the credibility of YouTube as a platform for health education, but also supports the effective management of NAFLD by improving public access to accurate dietary advice.

This study underscores the dual challenge of ensuring high-quality health information while maximizing engagement on digital platforms like YouTube. The findings highlight the need for collaborations between healthcare professionals and content creators to produce scientifically rigorous yet relatable content. By adopting narrative techniques and enhancing regulatory oversight, the quality and accessibility of online dietary guidance can be improved, ultimately supporting better health outcomes for individuals managing NAFLD.

## Figures and Tables

**Table 1:** Descriptive characteristics of YouTube videos discussing dietary recommendations for fatty liver disease management.

Video Features	All (n = 183)	Healthcare Professionals (n = 85)	Patients (n = 88)	Undefined (n = 10)	*p*-Value
Views	379,012 (12,100–1,200,000)	450,050 (23,000–1,800,000)	340,000 (15,000–1,000,000)	190,000 (10,000–700,000)	0.032
Likes	1512 (500–3700)	1680 (600–4100)	1400 (400–2900)	920 (300–2300)	0.045
Dislikes	75 (10–200)	89 (15–220)	60 (5–150)	50 (10–130)	0.091
Duration (min)	8.9 (5.2–13.8)	7.5 (4.8–10.5)	9.5 (5.9–14.7)	6.3 (3.5–9.2)	0.021
Subscriptions	19,800 (1200–45,000)	25,000 (3000–50,000)	15,000 (800–30,000)	12,000 (500–28,000)	0.012
Days Since Upload	980 (530–1400)	1150 (700–1800)	850 (400–1200)	710 (350–900)	0.001
Viewing Rate	4.8 (1.5–9.5)	3.9 (1.2–7.8)	5.2 (1.8–10.2)	4.3 (1.0–8.0)	0.063
Interaction Rate	1.8 (0.6–3.0)	1.5 (0.5–2.5)	2.1 (0.8–3.5)	1.3 (0.4–2.1)	0.028
Retention Rate (%)	60 (40–80)	67 (50–85)	54 (35–70)	50 (30–60)	0.015
Time Spent (min)	6.5 (3.0–10.0)	8.0 (4.5–12.0)	5.5 (3.0–8.0)	4.0 (2.0–5.5)	0.027
Scientific Evidence	60/183 (33%)	50/85 (59%)	8/88 (9%)	2/10 (20%)	<0.001
No Evidence	123/183 (67%)	35/85 (41%)	80/88 (91%)	8/10 (80%)	<0.001

Values are presented as median (interquartile range) or n/n (%). *p*-values represent differences among categories (Kruskal–Wallis test).

**Table 2:** Collected data and parameters evaluated for selected videos.

Video Characteristics	Parameters
Video details	Video Title, URL, Source, Upload date, Days online, and Video duration (min).
Metrics	Views, Likes, Dislikes, Subscriptions, Viewing rate, and Interaction rate.
Video quality	Image quality (poor, good, high-definition).
Educational content	Presence of diet recommendations, Clarity of audio commentary, and Clarity of written commentary.
DISCERN Score	Total DISCERN score and sub-scores (S1.1 to S2.15).
Diet Class	Categories of diet recommendations (e.g., “low-carb”, “keto”, “vitamin-rich”, etc.).
Overall assessment	General Quality Score (GQS), VIQI criteria (VIQI1 to VIQI4).

## Data Availability

The original contributions presented in this study are included in the article. Further inquiries can be directed to the corresponding author.
